# The Teeth

**Published:** 1892-04

**Authors:** 


### THE TEETH.

The proper names or designation of the teeth may be learned by a child in five minutes, yet a multitude of generally intelligent people go through life with no better method of designating any particular member of the dental family than by opening the mouth and placing the end of a finger upon the offender. Beginning at the centre of the adult jaw, the mouth which is fully equipped contains four sets of eight teeth each ; and as these sets correspond, one side of either jaw may be taken as an object lesson. Each set contains two incisors, one cuspid, two bicuspids and three molars, in the order named, beginning at the front. The first incisor is known as the central, the second as the lateral ; the cuspid, if in the upper jaw, is familiarly known as an "eye-tooth," in the lower jaw, as a "stomach-tooth." The bicuspids are simply called the first and second ; while the molars are known as the "six-year," "twelve-year," and "wisdom teeth," respectively. Add the designations, right or left, upper or lower, and any tooth can be instantly and unmistakably specified. A half-dozen other semi-technical terms in this connection may be frequently found useful. The labial surface of the teeth is that toward the lips ; the buccal, that facing the cheek ; the lingual, that next the tongue on the lower jaw ; the palatal, that facing the roof of the mouth. The approximal surfaces are those facing neighboring teeth ; of these the distal being those facing from the centre, the mesial those looking toward the centre of the jaw.

